# The Impact of Proximal Nasolacrimal Abnormalities on the Surgical Outcomes of Punctal Stenosis

**DOI:** 10.1155/joph/7692939

**Published:** 2026-07-09

**Authors:** Emre Ayintap, Mehmet Ali Doran, Hasan Aytogan

**Affiliations:** ^1^ Department of Ophthalmology, Republic of Türkiye Ministry of Health Izmir City Hospital, Bayrakli District Sevket Ince Neighborhood, Izmir, 35540, Türkiye; ^2^ Department of Ophthalmology, Republic of Türkiye Ministry of Health Midyat State Hospital, Bahcelievler Hastane Street, Midyat, 47500, Mardin, Türkiye; ^3^ Department of Ophthalmology, Republic of Türkiye Health Sience University Izmir Tepecik Training and Research Hospital, Gaziler Street No: 468 Yenişehir Konak, Izmir, 35120, Türkiye

**Keywords:** canalicular obstruction, canalicular pathology, epiphora, lacrimal drainage system, punctal stenosis, punctoplasty, surgical outcomes

## Abstract

**Purpose:**

Punctoplasty is the standard surgical treatment for punctal stenosis; however, underlying canalicular abnormalities may significantly impact functional outcomes. This study evaluates the frequency and nature of proximal canalicular pathologies in patients with severe punctal stenosis (Grade 0 or 1) undergoing snip punctoplasty without stent or plug implantation and explores their relationship with surgical success.

**Methods:**

This retrospective study included patients with Grade 0 or 1 punctal stenosis who underwent snip punctoplasty at Izmir Tepecik Training and Research Hospital between 2017 and 2023. Patients with systemic diseases, prior lacrimal surgeries, or nasolacrimal duct obstructions beyond the common were excluded. Punctoplasty was performed using the three‐snip technique without the use of punctal plugs or Mini‐Monoka stents. Intraoperative probing and lacrimal lavage were used to evaluate canalicular patency.

**Results:**

A total of 312 patients (516 eyes) were included. Surgical success was achieved in 396 eyes (76.74%). Failure occurred in 120 eyes (23.26%), with proximal canalicular stenosis being the most common associated pathology (38.33%), followed by mid‐canalicular (26.67%) obstructions. Since we exclusively included patients with Grade 0‐1 punctal stenosis, the relatively lower functional success observed in our cohort may reflect a higher underlying prevalence of canalicular pathology in this subgroup. This supports the hypothesis that advanced punctal stenosis is frequently accompanied by additional proximal lacrimal abnormalities, potentially contributing to surgical failure. Failure was significantly associated with increasing age, especially in patients ≥ 60 years (28.7%) compared to patients < 60 years (15.2%). Gender was not a significant factor.

**Conclusions:**

Although anatomical patency can be achieved with snip punctoplasty, functional success is often compromised by unrecognized canalicular abnormalities—particularly in patients with severe punctal stenosis. Chronic ocular inflammation and fibrosis may affect both the punctum and canalicular pathway. Adjunctive use of Mini‐Monoka stents should be considered to address deeper canalicular disease. Future prospective studies with standardized symptom scores and extended follow‐up are warranted.

## 1. Introduction

The lacrimal drainage system plays a critical role in maintaining ocular surface health by facilitating the physiologic drainage of tears from the ocular surface into the nasal cavity. This system is composed of several anatomical segments including the puncta, vertical and horizontal canaliculi, the common canaliculus, the lacrimal sac, and the nasolacrimal duct. Proper function of each component is necessary to ensure efficient tear drainage and ocular surface homeostasis.

Dysfunction may occur at any level of the lacrimal drainage pathway and may result from inflammatory processes, fibrosis, infection, trauma, medication toxicity, or age‐related degenerative changes. Obstruction of the distal nasolacrimal duct is a well‐recognized cause of epiphora; however, abnormalities involving the proximal lacrimal drainage system are increasingly recognized as important contributors to persistent tearing. Pathology affecting the punctum or canaliculi may impair tear drainage even when distal structures remain patent.

The puncta represent the entry point to the lacrimal drainage system and are located at the medial eyelid margin. Stenosis or complete occlusion of these structures may result in symptomatic epiphora and reduced quality of life. Chronic blepharitis, inflammation, trauma, systemic medications, and aging have all been implicated as potential etiological factors in the development of punctal stenosis.

Snip punctoplasty remains the most widely used surgical treatment for advanced punctal stenosis and aims to enlarge the punctal opening to restore tear outflow [1]. Although anatomical success rates following punctoplasty are generally high, functional outcomes may be less favorable when additional abnormalities exist within the proximal lacrimal system. Canalicular disease may remain undetected prior to surgery and can contribute to persistent postoperative epiphora.

The proximal lacrimal system includes the vertical and horizontal canaliculi and extends to the common canaliculus [2, 3]. Understanding the prevalence and distribution of canalicular abnormalities in patients with severe punctal stenosis may help improve preoperative evaluation strategies and guide surgical decision‐making. The aim of this study was therefore to investigate the relationship between proximal nasolacrimal abnormalities and functional outcomes following snip punctoplasty.

## 2. Methods

This retrospective study was conducted in accordance with the Declaration of Helsinki and approved by the Ethics Committee of Izmir City Hospital (Approval No: 2025/104; Approval date: 26 February 2025). All patients provided informed consent for the use of their anonymized clinical data.

**FIGURE 1:**
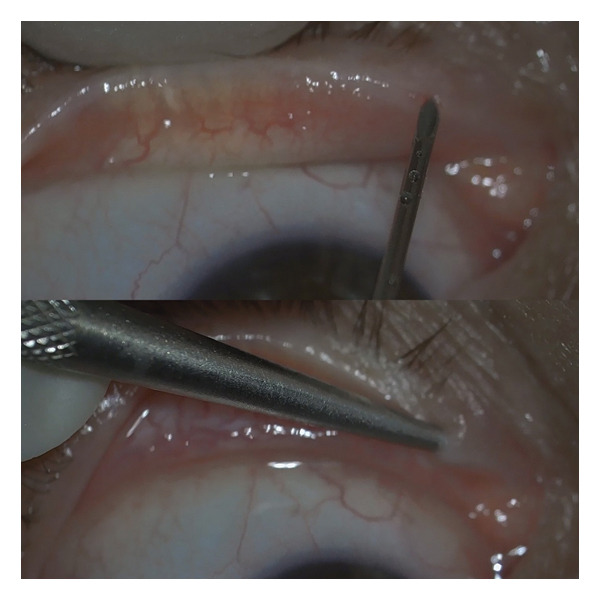
The punctal opening was found with a needle and dilated with a dilator.

Preoperative lacrimal system evaluation was routinely performed whenever technically feasible. Lacrimal syringing and probing were carried out preoperatively in patients with Grade 1 punctal stenosis in order to evaluate canalicular patency and localize the level of obstruction. In cases of Grade 0 stenosis (invisible punctum), preoperative irrigation was not possible because the punctal opening could not be identified. In these patients, the punctum was first identified intraoperatively using a needle, followed by dilation and probing with irrigation to evaluate canalicular patency (Figure 1).

Patients who had distal nasolacrimal duct obstruction beyond the common canaliculus, previous lacrimal surgery, systemic autoimmune disease, or recent chemotherapy were excluded from the study.

We retrospectively analyzed the records of patients who underwent snip punctoplasty for Grade 0 (invisible punctum) or Grade 1 (severely narrowed punctum) stenosis at the Oculoplasty Unit of Izmir Tepecik Training and Research Hospital between 2017 and 2023.

All surgeries were performed under local anesthesia using the standard three‐snip punctoplasty technique. The punctal opening was first identified using a fine needle and subsequently dilated with a punctal dilator. Vertical and horizontal incisions were then created to enlarge the punctal opening. Hemostasis was achieved with gentle pressure, and punctal patency was confirmed using a lacrimal probe.

Intraoperative assessment included lacrimal probing and irrigation with physiological saline in order to determine canalicular patency and localize the level of obstruction. Canalicular abnormalities were categorized based on probing length as proximal (< 4 mm), mid‐canalicular (4–8 mm), distal (> 8 mm), or common canalicular resistance [3–5].

When canalicular stenosis or resistance was encountered intraoperatively, gentle probing and irrigation were attempted to relieve membranous obstruction whenever possible. No silicone intubation or stenting procedures were performed, and all eyes included in the study underwent punctoplasty alone.

Functional failure was defined as persistence of symptomatic epiphora despite anatomically patent punctum following surgery.

Statistical analyses included independent sample *t*‐tests, chi‐square tests for categorical variables, and Mann–Whitney *U* tests for nonparametric distributions. Normality was assessed using the Kolmogorov–Smirnov test. A *p* value < 0.05 was considered statistically significant.

These methodological clarifications were incorporated during the revision process in response to reviewer comments in order to improve the transparency of the preoperative evaluation protocol. To evaluate the potential impact of intereye correlation in bilateral cases, an additional sensitivity analysis was performed including one eye per patient only. For patients who underwent bilateral surgery, one eye was selected according to a predefined laterality‐based approach for secondary analysis.

## 3. Results

This study was conducted to evaluate the clinical success rates, demographic distributions, presence of additional canalicular pathology, and the effect of age on these factors of punctoplasty operations applied to 312 patients. The data were analyzed retrospectively. The postoperative follow‐up duration ranged from 3 to 15 months, with a mean follow‐up of approximately 6 months. Functional outcomes were assessed at the final follow‐up visit. Functional failure was defined as persistence of symptomatic epiphora despite anatomically patent punctum after surgery.

About 64.1% (*n* = 200) of these patients were female and 35.9% (*n* = 112) were male. The mean age of female patients was calculated as 62.96 ± 9.12 years, and the mean age of male patients was calculated as 64.30 ± 8.56 years. The general mean age of all patients was 63.45 ± 8.97 years. No statistically significant difference was found between the mean ages (*t* = −1.237; *p* = 0.217).

Two hundred and four (65.38%) patients underwent bilateral procedures and 108 (34.62%) underwent unilateral procedures. The mean age of bilateral patients was 63.46 years, while the mean age of unilateral patients was 63.41 years, and no significant difference was found between the two groups (*t* = 0.051; *p* = 0.959). In the operations performed on a total of 516 eyes, successful results were obtained in 396 eyes (76.74%) (Table 1).

**TABLE 1:** Demographic characteristics and surgical success.

Characteristic	Value
Total number of patients	312
Total number of eyes	516
Female	200 (64.1%)
Male	112 (35.9%)
Mean age (years)	63.45 ± 8.97
Bilateral surgery	204 patients (65.38%)
Unilateral surgery	108 patients (34.62%)
Successful eyes	396 (76.74%)
Eyes with functional failure	120 (23.26%)

**FIGURE 2:**
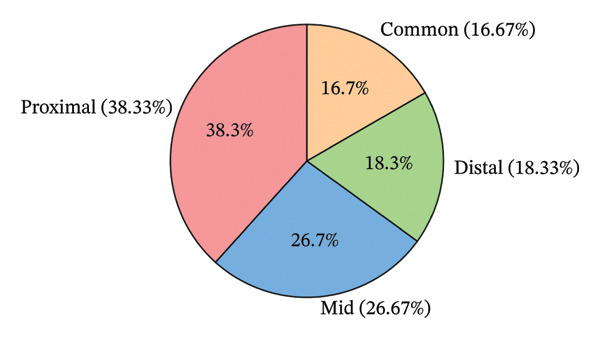
Distribution of canalicular pathologies (pie chart).

Among additional canalicular pathologies, proximal canalicular obstruction was detected at 38.33% (48 eyes), mid‐canalicular obstruction at 26.67% (32 eyes), distal canalicular obstruction at 18.33% (22 eyes), and common canalicular obstruction at 16.67% (18 eyes) (Figure 2).

The relationship between gender and additional canalicular pathologies was evaluated with the chi‐square test. According to the test results, the distribution of proximal, mid, distal, and common canalicular obstructions between women and men did not show a statistically significant difference (Chi2 = 7.85; *p* = 0.097). This result shows that gender has no significant effect on additional canalicular pathology types. The difference between age groups and additional pathologies was not found to be statistically significant (Chi2 = 5.942; *p* = 0.114).

The relationship between failure in punctoplasty operations and the ages of the patients was statistically examined. The average age of the eyes with unsuccessful results was calculated as 66.74 years, and the average age of the eyes with successful results was calculated as 62.91 years. According to the *t*‐test results performed between the groups, a statistically significant relationship was found between age and failure (*t* = 3.214; *p* = 0.001). This result shows that the failure rate may increase with age.

**FIGURE 3:**
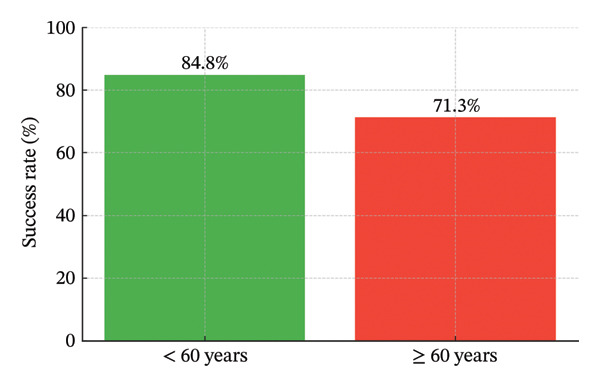
Surgical success rate by age group (bar graph).

In the subanalysis based on age groups, the failure rate was found to be 15.2% in patients under 60 years of age and 28.7% in patients 60 years of age and older. The difference between these groups was statistically significant (Chi2 = 8.475; *p* = 0.004). This result supports that increasing age may be an important factor that increases failure rates (Figure 3). To address potential clustering bias related to bilateral cases, a sensitivity analysis including one eye per patient was performed. In this secondary analysis, the previously observed association between increasing age and surgical failure was no longer statistically significant (mean age in failure group: 63.54 years vs. 63.41 years in the success group, *p* = 0.921).

## 4. Discussion

Punctal stenosis is a common disorder of the proximal lacrimal tract, accounting for 3%–17% of all lacrimal drainage disorders in large epidemiology studies [6, 7]. This study highlights the frequent coexistence of canalicular abnormalities in patients undergoing punctoplasty for advanced punctal stenosis. While anatomical success is typically high following snip punctoplasty, functional outcomes may lag due to proximal obstructions that persist beyond the punctum. The present findings emphasize the importance of careful evaluation of the proximal lacrimal drainage system in patients presenting with severe punctal stenosis. In routine clinical practice, punctal pathology may coexist with unrecognized canalicular abnormalities, which may partially explain persistent postoperative epiphora despite technically successful punctoplasty.

Although increasing age was associated with surgical failure in the primary eye‐based analysis, this association was not maintained in sensitivity analysis restricted to one eye per patient. This suggests that the observed relationship may have been influenced by intereye correlation in bilateral cases. Therefore, the role of age as an independent predictor of punctoplasty failure should be interpreted cautiously and requires further investigation in prospective studies. Chronic ocular surface inflammation, including blepharitis, may exacerbate this process, suggesting the need for perioperative management of inflammation. The etiological factors of punctal stenosis and obstruction have been described in various reports, including blepharitis, tissue atrophy, ectropion, systemic 5‐FU chemotherapy, and antiglaucoma eye drops [8–10].

Chronic blepharitis is a well‐known predisposing factor in cases of punctal stenosis [11, 12]. Several additional factors may influence functional outcomes after punctoplasty, including eyelid laxity, medial canthal tendon status, orbicularis oculi function, and lacrimal pump efficiency. These variables were not systematically evaluated in the present retrospective study and may therefore represent potential confounding factors.

In our study, chronic blepharitis and inflammation were observed as predisposing factors.

Punctoplasty is the main treatment for punctum stenosis. Anatomical and functional success varies between 18.7% and 93.3% [13–19]. The lack of significant gender differences in outcomes aligns with the existing literature. However, the high rate of proximal and mid‐canalicular involvement—especially in Grade 0‐1 stenosis—suggests that punctal disease is often a marker of more extensive lacrimal system dysfunction.

The use of probing and lavage intraoperatively proved valuable in detecting hidden obstructions. In patients with suspected canalicular disease, primary snip punctoplasty may be inadequate. In such cases, stent placement (e.g., Mini‐Monoka) or combined procedures may offer better functional relief [20, 21].

Moreover, progressive dysfunction of the canaliculi, impairment of tear flow, and collapse of the tear duct may contribute to postoperative complications. These factors contribute to the failure of punctoplasty surgery.

To enhance the success rate of punctoplasty, appropriate interventions should be implemented to optimize the patient’s ocular health before and after surgery. Effective management of chronic ocular inflammatory conditions and systematic assessment of the lacrimal drainage system can help mitigate postoperative complications. Stents may serve as either an alternative to punctoplasty or an adjunctive measure.

This study has several limitations. First, its retrospective design introduces inherent risks of selection bias and incomplete data collection compared with prospective investigations. Second, the absence of standardized objective functional outcome measures such as the Munk score or fluorescein dye disappearance testing limits the precision of postoperative functional assessment. Third, potentially relevant confounding variables including eyelid laxity, medial canthal tendon integrity, and lacrimal pump function were not systematically documented in the retrospective dataset and therefore could not be analyzed. In addition, a substantial proportion of patients underwent bilateral surgery. Although sensitivity analysis restricted to one eye per patient was performed, inclusion of bilateral cases in the primary eye‐based analysis may still have influenced some statistical associations. Future prospective studies incorporating standardized functional testing and comprehensive periocular assessment are warranted to validate these findings.

## 5. Conclusions

Snip punctoplasty yields high anatomical success in Grade 0‐1 punctal stenosis but has limited functional success when unrecognized canalicular pathologies are present. Intraoperative probing and lavage can identify such issues. Further studies are needed to clarify patient‐related factors associated with functional failure following punctoplasty. Future studies may investigate whether adjunctive interventions such as silicone stenting improve outcomes in selected patients with concomitant canalicular abnormalities.

## Author Contributions

Emre Ayintap, Mehmet Ali Doran, and Hasan Aytogan participated in the surgeries and contributed to data collection, data analysis, and writing of the manuscript.

## Funding

The authors declare that they have not received any funding for this study.

## Disclosure

All authors read and approved the final manuscript.

## Ethics Statement

This study was approved by the Ethical Committee of Izmir City Hospital. This retrospective chart review study was in accordance with the 1964 Helsinki Declaration and its later amendments or comparable ethical standards. Individual written informed consent was obtained. Ethics committee approval number: 2025/104. Approval date: 26/02/2025.

## Consent

A written informed consent was taken from patients for publication.

## Conflicts of Interest

The authors declare no conflicts of interest.

## Data Availability

The datasets used and/or analyzed during the current study are available from the corresponding author on reasonable request.

## References

[1] Soiberman U. , Kakizaki H. , Selva D. , and Leibovitch I. , Punctal Stenosis: Definition, Diagnosis, and Treatment, Clinical Ophthalmology. (2012) 6, 1011–1018, 10.2147/opth.s31904. 22848141 PMC3402122

[2] Liarakos V. S. , Boboridis K. G. , Mavrikakis E. , and Mavrikakis I. , Management of Canalicular Obstructions, Current Opinion in Ophthalmology. (2009) 20, no. 5, 395–400, 10.1097/icu.0b013e32832ec3e0. 19587600

[3] Welham R. A. and Henderson P. , Results of Dacryocystorhinostomy Analysis of Causes for Failure, Transactions of the Ophthalmological Societies of the United Kingdom. (1973) 93, 601–609. 4602178

[4] Boboridis K. G. , Bunce C. , and Rose G. E. , Outcome of External Dacryocystorhinostomy Combined with Membranectomy of a Distal Canalicular Obstruction, American Journal of Ophthalmology. (2005) 139, no. 6, 1051–1055, 10.1016/j.ajo.2005.01.009. 15953435

[5] Starks V. S. and Yoon M. K. , Acquired Obliteration of the Proximal Lacrimal Drainage System, Ophthalmic Plastic and Reconstructive Surgery. (2019) 35, no. 4, 342–345, 10.1097/iop.0000000000001244. 30365474

[6] Ali M. J. , Malhotra R. , and Patel B. C. , Routine Punctoplasty: isn’t It Time We Preserved the Integrity of the Punctum?, Orbit. (2022) 41, no. 4, 407–412, 10.1080/01676830.2022.2055087. 35502152

[7] Viso E. , Rodriguez-Ares M. T. , and Gude F. , Prevalence and Associations of External Punctal Stenosis in a General Population in Spain, Cornea. (2012) 31, no. 11, 1240–1245, 10.1097/ico.0b013e31823f8eca. 22367046

[8] Hur M. C. , Jin S. W. , Roh M. S. et al., Classification of Lacrimal Punctal Stenosis and Its Related Histopathological Feature in Patients with Epiphora, Korean Journal of Ophthalmology. (2017) 31, no. 5, 375–382, 10.3341/kjo.2016.0129. 28994268 PMC5636712

[9] Fezza J. P. , Wesley R. E. , and Klippenstein K. A. , The Treatment of Punctal and Canalicular Stenosis in Patients on Systemic 5-FU, 1999, SLACK Incorporated Thorofare, 105–108. 10037204

[10] McNab A. A. , Lacrimal Canalicular Obstruction Associated with Topical Ocular Medication, Australian and New Zealand Journal of Ophthalmology. (1998) 26, no. 3, 219–223, 10.1111/j.1442-9071.1998.tb01315.x. 9717753

[11] Kashkouli M. B. , Beigi B. , Murthy R. , and Astbury N. , Acquired External Punctal Stenosis: Etiology and Associated Findings, American Journal of Ophthalmology. (2003) 136, no. 6, 1079–1084, 10.1016/s0002-9394(03)00664-0. 14644218

[12] Edelstein J. and Reiss G. , The Wedge Punctoplasty for Treatment of Punctal Stenosis, 1992, SLACK Incorporated Thorofare, 818–821. 1494436

[13] Chak M. and Irvine F. , Rectangular 3-Snip Punctoplasty Outcomes: Preservation of the Lacrimal Pump in Punctoplasty Surgery, Ophthalmic Plastic and Reconstructive Surgery. (2009) 25, no. 2, 134–135, 10.1097/iop.0b013e3181994062. 19300158

[14] Cao X. , Hu Z.-Z. , Wu Y. , Song Y. , and Liu Q.-H. , Rectangular 3-Snip Punctoplasty Versus Punch Punctoplasty with Silicone Intubation for Acquired External Punctal Stenosis: A Prospective Randomized Comparative Study, International Journal of Ophthalmology. (2021) 14, no. 6, 849–854, 10.18240/ijo.2021.06.09. 34150539 PMC8165618

[15] Murdock J. , Lee W. W. , Zatezalo C. C. , and Ballin A. , Three-Snip Punctoplasty Outcome Rates and Follow-Up Treatments, Orbit. (2015) 34, no. 3, 160–163, 10.3109/01676830.2015.1014513. 25906237

[16] Chalvatzis N. T. , Tzamalis A. K. , Mavrikakis I. , Tsinopoulos I. , and Dimitrakos S. , Self-Retaining Bicanaliculus Stents as an Adjunct to 3-snip Punctoplasty in Management of Upper Lacrimal Duct Stenosis: a Comparison to Standard 3-snip Procedure, Ophthalmic Plastic and Reconstructive Surgery. (2013) 29, no. 2, 123–127, 10.1097/iop.0b013e31827f5a10. 23392314

[17] Park S. J. , Noh J. H. , Park K. B. , Jang S. Y. , and Lee J. W. , A Novel Surgical Technique for Punctal Stenosis: Placement of Three Interrupted Sutures After Rectangular three-snip Punctoplasty, BMC Ophthalmology. (2018) 18, no. 1, 10.1186/s12886-018-0733-2. PMC5836447 29506497

[18] Makselis A. , Petroska D. , Kadziauskiene A. et al., Acquired Nasolacrimal Duct Obstruction: Clinical and Histological Findings of 275 Cases, BMC Ophthalmology. (2022) 22, no. 1, 10.1186/s12886-021-02185-x. PMC8734260 34986808

[19] Park J. W. , Han J. , Choi W. K. , Kim J. , and Choi C. Y. , Simple Surgical Punctal Occlusion with High Frequency Radiowave Electrosurgery, BMC Ophthalmology. (2023) 23, no. 1, 10.1186/s12886-023-02798-4. PMC9893631 36732735

[20] Hussain R. , Kanani H. , and McMullan T. , Use of Mini-Monoka Stents for Punctal/Canalicular Stenosis, British Journal of Ophthalmology. (2012) 96, no. 5, 671–673, 10.1136/bjophthalmol-2011-300670. 22241928

[21] Azzaro C. , Meduri A. , Oliverio G. W. et al., The Use of Venous Catheter and Irrigation With Povidone-Iodine 0.6% in Patients With Punctal and Proximal Canalicular Stenosis: Preliminary Report, Journal of Clinical Medicine. (2024) 13, no. 5, 10.3390/jcm13051330. PMC10932350 38592157

